# Image Matching for UAV Geolocation: Classical and Deep Learning Approaches

**DOI:** 10.3390/jimaging11110409

**Published:** 2025-11-12

**Authors:** Fatih Baykal, Mehmet İrfan Gedik, Constantino Carlos Reyes-Aldasoro, Cefa Karabağ

**Affiliations:** 1 Department of Unmanned Aerial Vehicle Technology and Operator, Vocational School of Higher Education, OSTIM Technical University, 06374 Ankara, Türkiye; 2 Department of Electric, Vocational School of Higher Education, OSTIM Technical University, 06374 Ankara, Türkiye; mehmetirfan.gedik@ostimteknik.edu.tr; 3 Department of Computer Science, School of Science & Technology, City St George’s, University of London, London EC1V 0HB, UK; constantino-carlos.reyes-aldasoro@citystgeorges.ac.uk

**Keywords:** GPS-Free Positioning, LOFTR, SIFT, AKAZE, SuperPoint + SuperGlue, NCC + Voting

## Abstract

Today, unmanned aerial vehicles (UAVs) are heavily dependent on Global Navigation Satellite Systems (GNSSs) for positioning and navigation. However, GNSS signals are vulnerable to jamming and spoofing attacks. This poses serious security risks, especially for military operations and critical civilian missions. In order to solve this problem, an image-based geolocation system has been developed that eliminates GNSS dependency. The proposed system estimates the geographical location of the UAV by matching the aerial images taken by the UAV with previously georeferenced high-resolution satellite images. For this purpose, common visual features were determined between satellite and UAV images and matching operations were carried out using methods based on the homography matrix. Thanks to image processing, a significant relationship has been established between the area where the UAV is located and the geographical coordinates, and reliable positioning is ensured even in cases where GNSS signals cannot be used. Within the scope of the study, traditional methods such as SIFT, AKAZE, and Multiple Template Matching were compared with learning-based methods including SuperPoint, SuperGlue, and LoFTR. The results showed that deep learning-based approaches can make successful matches, especially at high altitudes.

## 1. Introduction

Today, unmanned aerial vehicles (UAVs) are actively involved in many areas such as reconnaissance [1], surveillance [2], search and rescue [3] and border security. The successful execution of such missions depends on the extremely precise determination of the location of the UAV. In current applications, this need is generally met through Global Navigation Satellite Systems (GNSSs); however, alternative positioning approaches such as inertial navigation [4] , visual odometry [5] , and image-based [6] geolocation have also been investigated. Nevertheless, GNSS signals are susceptible to both natural environmental obstacles and intentional interference due to their low transmission power. For example, GPS spoofing attacks can cause the UAV to head to the wrong location, causing serious mission losses [7]. On the other hand, jamming attacks can completely intercept the GNSS signal, eliminating the vehicle’s ability to fly autonomously. The operational effects of such threats, especially in military operations and critical civilian missions, are extremely serious. Therefore, the development of alternative positioning methods that will reduce or completely eliminate the dependence on GNSS systems is of great strategic importance [8]. In fact, previous studies have highlighted that GNSS receivers face technical limitations in indoor or obstructed environments [9], and recent works emphasize the growing role of UWB or multisource fusion methods to enhance robustness in such conditions [10,11].

In environments where GNSS signals lose their reliability, the ability of UAVs to determine their positions based only on visual data has become an important field of study that has attracted great attention from researchers in recent years [12,13,14]. In this context, various approaches that combine different data sources or use deep learning-based map querying methods have been proposed. For example, in some studies, location estimation could be made without GNSS using only drone camera and IMU data; for this purpose, visual-inertial odometry (VIO) and visual location recognition (VPR) were evaluated together [15]. Early visual navigation systems demonstrated the feasibility of autonomous UAV operation based on monocular camera data [16], and subsequent research achieved autonomous flight in unknown indoor environments using visual perception alone [17]. Similarly, systems that jointly optimize image retrieval and local matching steps have been shown to be able to perform drone positioning over large areas in seconds and with an accuracy of several meters [18]. Moreover, multimodal approaches that fuse infrared and RGB cameras have been applied to UAV pose estimation with promising results [19], while precision landing solutions using visual or IR marker patterns have also been developed [20,21]. In parallel, lightweight IMU-based algorithms [22] and advanced filtering techniques such as EKF and UKF have been employed to improve robustness in localization and state estimation [23,24,25].

In real-world UAV operations, recent research has introduced real-time aerial image registration techniques built upon traditional feature matching strategies [26]. At the same time, several studies have turned their attention toward closing the gap between classical detector–descriptor pipelines and modern dense learning-based matchers, aiming to enhance both robustness and geometric consistency [27].

Although these studies provide valuable progress, most lack analyses grounded in geometric transformation models and seldom include systematic evaluations of homography-based satellite–drone alignment. These shortcomings make it difficult to evaluate the consistency and practical applicability of the developed methods in real field conditions.

This study, on the other hand, aims to provide a comparative analysis on the accuracy of direct homography conversion by testing satellite–drone image (representative) matching, which is the basic building block of a positioning system that is not dependent on GNSS, with both traditional and deep learning-based algorithms. Thus, by measuring the performances of different methods in field applications, both explanatory and comparative contributions are made to the visual positioning literature.

## 2. Materials and Methods

In this study, JPEG format images obtained from different altitudes were prepared using Google Earth Pro (v7.3.6.10441) software to test the accuracy of image matching algorithms. First, the representative drone image, supposedly obtained from an altitude of 150 m, was exported at a resolution of 8192×4320 pixels (8 K Ultra High Definition) to cover the target area. Then, this image was transferred to the working environment with the help of QGIS 3.42.2 software and subjected to georeferencing.

Using the “Georeferencing Tool” in the QGIS software, the latitude and longitude information of the four corner points of the image were obtained from the Google Earth environment in DMS (Degree-Minute-Second) format. This data was converted to Decimal Degree format and introduced to the software. The Helmert transform [28] was preferred as the transformation method, and the target coordinate reference system was determined as EPSG:4326 (WGS 84). The Lanczos (6×6 Kernel) interpolation algorithm was used for resampling. The georeferencing process results in a GeoTIFF-formatted image file that can be used directly in spatial analyses, with each pixel in the image matched to real-world coordinates, geographically accurately located.

A similar process was applied to a total of ten satellite images representing different altitudes, starting at 500 m and increasing at intervals of 500 m up to 5000 m. These images were also exported in JPEG format via the Google Earth Pro v7.3.6.10441 application and georeferencing was performed using the same transformation parameters (Helmert transform, EPSG:4326, Lanczos interpolation) for each. Thus, the comparability of the performance of the matching algorithms on images with different resolutions is ensured.

The Google Earth data we use is not a raw satellite image, but has been compiled, processed, and orthorectified from different sources such as Airbus (for high resolution) and Landsat/Copernicus (for the base layer). One of the main providers of high-resolution data that Google Earth uses is Airbus. This data partnership between the two companies is also documented by Airbus’ establishment of its own satellite data distribution platform, OneAtlas, on Google Cloud infrastructure [29].

The main purpose of georeferencing operations is to determine the deviation between the location and the real world coordinates detected with the help of the matching algorithm on the representative drone image. The actual drone position was predetermined with the help of an external measurement system and used as reference data in the comparison process. Accordingly, the drone’s position on the real world is defined as 39.969539 degrees north latitude and 32.745036 degrees east longitude. The estimated coordinates obtained during the image matching process were compared with the previously known reference point. Thus, the position error was determined based on the distance between the estimated position and the actual location, and the accuracy of the georeferencing process was numerically evaluated using this error metric.

In this study, a method based on homography calculations was adopted along with a deep learning-based matching algorithm. An image was obtained from satellite imagery to simulate a drone perspective at an altitude of 150 m above the ground. All algorithms were run on the A100 GPU through the PyTorch library (2.1.0) in the Google Colab Pro+ environment.

The range of “500–5000” m is not the actual orbits of satellites, but the “virtual altitudes” used to simulate the different resolution levels of satellite images. The concept of assuming that the UAV image is obtained by vertical shooting and that the image center represents the projection of the UAV on the ground has been accepted. The “150” m altitude mentioned for the drone represents an approximate typical civilian UAV operation.

### 2.1. Classical Feature Matching Algorithms

In this study, three different methods based on traditional image processing algorithms are proposed to accurately determine the position of a representative drone image obtained from low altitude on georeferenced satellite images representing different altitude levels. The developed algorithms have three basic structures: feature-based matching (SIFT and AKAZE), geometric validation (USAC_MAGSAC algorithm), and template-based multiple matching (NCC + voting). Satellite and drone images in GeoTIFF format containing geolocation information and high dynamic range were used as input data in all methods.

### 2.2. Preparation of Images and Pre-Processing Steps

The satellite and drone images used in the study are in raster format (GeoTIFF) with .tif extension and geolocation information. These images were normalized in the first stage and made processable. The normalization process was performed with the following transformation, which allows the pixel density values to be scaled to the range of 0–255:

(1) $$I_{norm}\left(x,y\right)=255\cdot \frac{I\left(x,y\right)-I_{min}}{I_{max}-I_{min}}$$

where I(x,y) is the raw pixel value in the image; $I_{min}$ and $I_{max}$ refer to the minimum and maximum pixel values, respectively.

The CLAHE (Contrast Limited Adaptive Histogram Equalization) algorithm [30] was applied to increase local contrast on normalised images. Unlike classical histogram equalisation, CLAHE increases local contrast by dividing the image into small regions and limits noise in high-contrast regions [30].

### 2.3. Feature-Based Mapping Methods

#### 2.3.1. SIFT (Scale-Invariant Feature Transform) Based Approach

The SIFT [31] algorithm detects keypoints in scale-space and generates descriptor vectors for these points. In the first step of the algorithm, the scale space is created by applying Gaussian filtering [31]:

(2) $$L\left(x,y,\sigma \right)=G\left(x,y,\sigma \right)*I\left(x,y\right)\;\mathrm{and}\;G\left(x,y,\sigma \right)=\frac{1}{2\pi \sigma^{2}}e^{-\frac{x^{2}+y^{2}}{2\sigma^{2}}}$$

L(x,y,σ): Blurred image at scale parameter σ (scale-space representation);
G(x,y,σ): 2D Gaussian kernel with standard deviation σ;
I(x,y): Input (original) image;
∗: Convolution operator (filtering).

Extreme points are selected from DoG (Difference of Gaussians) images. The descriptor vectors for these points are 128-dimensional attributes derived from directional histograms [31]. The obtained descriptors were matched with the FLANN (Fast Library for Approximate Nearest Neighbors) algorithm [32] and the matching accuracy was increased by the Lowe’s ratio test.

#### 2.3.2. AKAZE (Accelerated-KAZE) Based Approach

The AKAZE [33] algorithm generates a multiscale representation using nonlinear diffusion equations. The basic mathematical model is expressed by the following differential equation:

(3) $$\frac{\partial L}{\partial t}=\nabla \cdot \left(c(x,y,t)\nabla L\right)$$

where c(x,y,t) is an edge-saver diffusion coefficient, which allows the high-frequency components in the image to be protected. The MLDB (Modified Local Difference Binary) method [33] is used in the production of descriptors.

### 2.4. Geometric Verification: Homography Matrix Calculation with USAC_MAGSAC

The matching points obtained by both methods were used to estimate $\left\{x_{i}↔x_{i}^{\prime }\right\}$ and homography matrix *H*. This transformation refers to the projective relationship in 2D space:

(4) $$x_{i}^{\prime }∼H\cdot x_{i}$$

The estimation of the homograph matrix was performed by the USAC_MAGSAC algorithm, which eliminated outliers [34].

### 2.5. Template-Based Mapping: Multiple NCC + Voting Approach

For situations where traditional matching methods may fail, a template-based method has been proposed. Three different variants (rotated, scaled, histogram equalised) derived from the drone image were searched separately in the satellite image. The NCC [35] (Normalized Cross-Correlation) similarity metric for each template is calculated as follows:

(5) $$NCC\left(x,y\right)=\frac{\sum_{i,j}\left[T\left(i,j\right)-\bar{T}\right]\left[I\left(x+i,y+j\right)-{\bar{I}}_{x,y}\right]}{\sqrt{\sum_{i,j}{\left[T\left(i,j\right)-\bar{T}\right]}^{2}\sum_{i,j}{\left[I\left(x+i,y+j\right)-{\bar{I}}_{x,y}\right]}^{2}}}$$

where *T* shows the template obtained from the drone image. *I* represents satellite imagery. The position with the highest correlation value is reflected as a vote on the voting map. The region with the most votes is determined as the final prediction point [35].

### 2.6. Geographical Coordinate and Distance Calculation

The obtained forecast coordinates were transferred to the WGS84 reference system with the help of affine conversion in the GeoTIFF file:

(6) $$\left[\begin{array}{c} Lon\\ Lat \end{array}\right]=Affine\cdot \left[\begin{array}{c} x\\ y\\ 1 \end{array}\right]$$

This affine transformation matrix is stored in the GeoTIFF file’s metadata and contains all the necessary parameters to convert pixel coordinates (x,y) into geographic coordinates (e.g., Longitude, Latitude, or UTM). The matrix is generally composed of six parameters that encode the following information:

- The real-world coordinate of the image’s origin (top-left pixel at (0,0)).
- The pixel size (scale or resolution) in the x and y directions.
- The orientation (rotation) of the image.

For instance, consider a simple affine transformation that converts the pixel coordinates ($x_{p},y_{p}$) of an image to geographic coordinates ($X_{geo},Y_{geo}$). The transformation matrix can be defined as follows:

$$\left[\begin{array}{c} X_{geo}\\ Y_{geo} \end{array}\right]=\left[\begin{array}{ccc} a & b & c\\ d & e & f \end{array}\right]\left[\begin{array}{c} x_{p}\\ y_{p}\\ 1 \end{array}\right]$$

where

- *c* and *f* are the *X* and *Y* coordinates of the top-left pixel, respectively.
- *a* and *e* represent the resolution of a pixel in the x and y directions (e.g., meters/pixel).
- *b* and *d* are the rotation parameters (typically zero for north-aligned images).

The distance between these coordinates and the known GPS location of the drone is calculated by the following formula based on the Vincenty model [36]:

(7) $$d=a\cdot \arccos\left[\sin\phi_{1}\sin\phi_{2}+\cos\phi_{1}\cos\phi_{2}\cos\left(\lambda_{1}-\lambda_{2}\right)\right]$$

These values were used as the basic metric for numerically assessing match accuracy [36].

### 2.7. LoFTR: Detector-Free Local Feature Matching with Transformers, Super Point and Super Glue

In this study, the satellite and drone images were uploaded in gray scale format using the OpenCV library, and the drone image was rescaled to a fixed pixel size of 1024×1024. The satellite image is divided into parts (tile) so that each of them is 1024×1024 pixels in size. During the scanning process, each window was shifted by a 512-pixel step, thus providing 50% overlap, improving match performance and smoothing the transition between windows. For visualisation purposes, grid lines in purple have been added to the satellite image, with dimensions of only 512×512. Actual matching operations were performed on windows with 1024×1024 pixels each. The matching operations were carried out with the LoFTR model [37,38] in the Kornia feature module, which utilized the outdoor version pretrained on the MegaDepth dataset.

**Figure 1:**
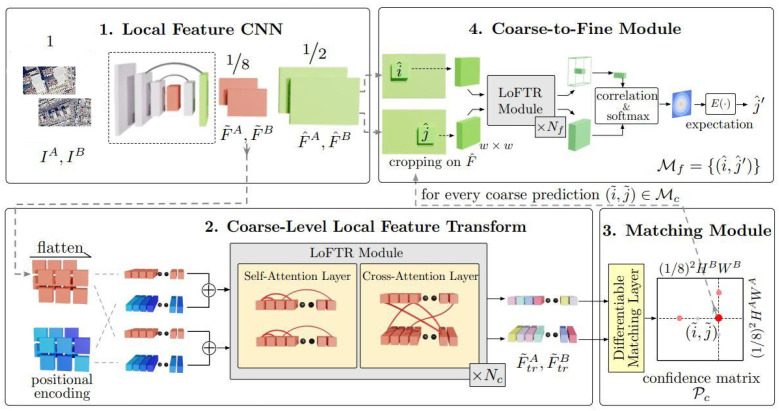
Overall architecture of the LoFTR (Local Feature TRansformer) network [37]. The framework consists of four main stages: (**1**) Local Feature CNN for extracting coarse and fine feature maps (${\hat{F}}^{A},\;{\hat{F}}^{B}$: fine-level future map; ${\tilde{F}}^{A},\;{\tilde{F}}^{B}$: coarse-level future map; $I^{A},\;I^{B}$: image pair); (**2**) Coarse-Level Local Feature Transform using self and cross-attention layers (${\tilde{F}}_{\mathrm{tr}}^{A},\;{\tilde{F}}_{\mathrm{tr}}^{B}$: transformed feature); (**3**) Differentiable Matching Module that generates a confidence matrix for coarse correspondences; (**4**) Coarse-to-Fine refinement to obtain sub-pixel-accurate feature matches.

The LoFTR (Local Feature TRansformer) network consists of 4 basic parts, as shown in (Figure 1). It has been developed to establish dense and accurate correspondences between two images. This architecture can operate without the need for explicit keypoint detection. First, a CNN is used, in which coarse features at 1/8 resolution capture the general structural context, and fine features at 1/2 resolution preserve detailed local textures. These multi-layer feature maps obtained from the CNN module are fed into the Local Feature Transform module. The self-attention layers in this section reinforce coherence by modeling the connections within the images themselves. Cross-attention layers, on the other hand, learn the relationships between the features of two different images and align these images in a common latent space.

After the alignment process, in which the representation is obtained at the contextual level, the module called the Differentiable Matching Module comes into play and calculates the similarities between the feature pairs and creates a confidence matrix. This matrix predicts which feature is most likely to match with each feature in the opposite image. The final part of the architecture is the Coarse-to-Fine improvement. It enables more precise updating of rough matches with the help of high-resolution feature maps. After calculating local similarities, it may be possible to achieve sub-pixel accuracy in final matches by using the softmax-based expectation method. LoFTR is an integrated architecture that combines convolutional encoding, attention-based alignment, differentiable matching, and coarse-to-fine hierarchical optimization steps [37].

**Figure 2:**
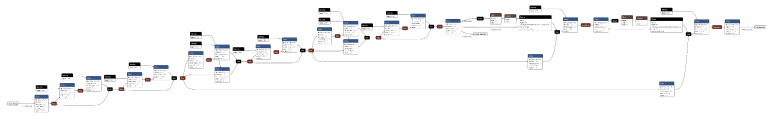
The LoFTR backbone architecture: a ResNet-FPN structure that extracts multi-scale feature maps by combining deep semantic and shallow positional information before feeding them into the transformer layers.

The backbone of LoFTR (Figure 2) uses the ResNet-FPN (Residual Network with Feature Pyramid Network) structure, which is a much more modern, deep and complex architecture compared to SuperPoint. The basis of this architecture is the ResNet body, which is built on “residual blocks”. The “skip connections” within these blocks allow the network to go much deeper, preventing issues such as gradient disappearance encountered during training and ensuring a more stable learning process. Once the image passes through this deep ResNet body, the second and most critical part of the architecture, the Feature Pyramid Network (FPN), comes into play. FPN retrieves feature maps of different scales obtained from different depths of ResNet (e.g., layer1, layer2, layer3). It combines semantically rich but low-resolution information from the deepest layer with positionally sensitive information from the shallower layers by carrying upward Interpolate (magnification) operations. This merging process produces two separate multi-scale and contextually rich feature maps, which are presented as input to the Transformer layers of LoFTR, accommodating both coarse (coarse features) and fine (fine features) details.

Z In addition to the LoFTR model, the same satellite and drone image dataset was also processed using the jointly applied SuperPoint [39] and SuperGlue [40] models to perform a comparative matching and localisation analysis.

The SuperPoint network [39] was used as a pretrained feature detector and descriptor in this study. The model was initially trained on the Synthetic Shapes dataset in a self-supervised manner through the MagicPoint stage, and subsequently fine-tuned on the MS-COCO dataset, while its performance was validated on the HPatches dataset. The SuperGlue network [40] was used in its pretrained outdoor version, trained on the MegaDepth dataset to perform feature correspondence matching between keypoints detected by SuperPoint using a graph neural network architecture.

**Figure 3:**
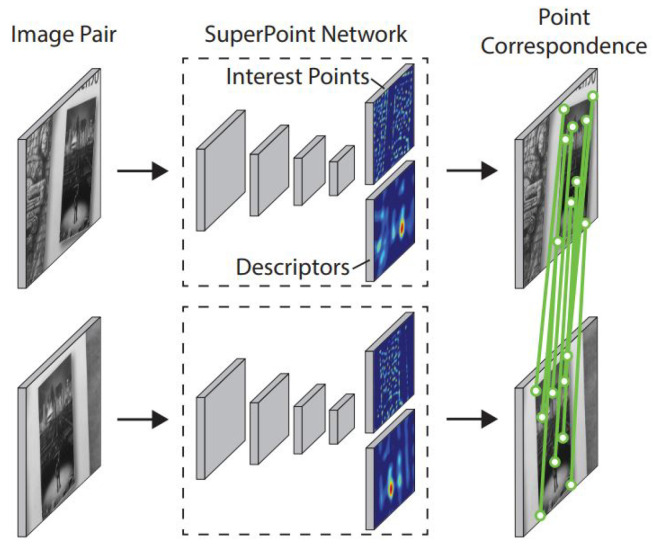
Structural overview of the SuperPoint network: the architecture in which keypoint detection and descriptor extraction processes are jointly performed within a unified convolutional framework [39].

The SuperPoint architecture is a fully convolutional neural network that detects interest points and generates local feature descriptors corresponding to these points from an input image. While traditional methods perform these two operations in separate stages, SuperPoint unifies them within a single framework, allowing for real-time and consistent feature extraction. As illustrated in (Figure 3), the detected keypoints are repeatable across different imaging conditions and correspond to distinctive regions in the scene, while the learned descriptors encode their local structural information. Thus, reliable geometric correspondences can be established between image pairs.

**Figure 4:**
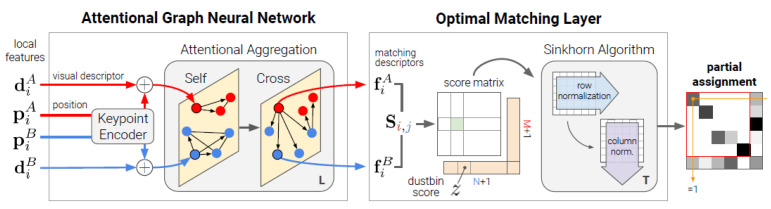
Structural overview of the SuperGlue architecture: a graph neural network-based matching framework that integrates attention mechanisms and optimal transport to establish reliable correspondences between image pairs [40].

As shown in (Figure 4), the SuperGlue architecture takes as input the keypoints and descriptors extracted by SuperPoint and consists of two key components: a Graph Neural Network (GNN) based on the attention mechanism and an Optimal Matching Layer. In the first stage, the GNN processes the keypoints and their visual descriptors through a positional encoder; it then alternately applies self-attention and cross-attention operations to integrate contextual information within the images themselves and between the two images. Through this structure, SuperGlue can reason about geometric relationships and eliminate ambiguities between visually similar regions. The Optimal Matching Layer in the second stage generates a similarity score matrix and computes a differentiable optimal transport solution using the Sinkhorn algorithm. As a result of this process, a soft and partial matching between keypoints is obtained. Thanks to this approach, SuperGlue can successfully learn reliable matches while filtering out unsuitable correspondences and performs much better than traditional heuristic-based matchers.

**Figure 5:**
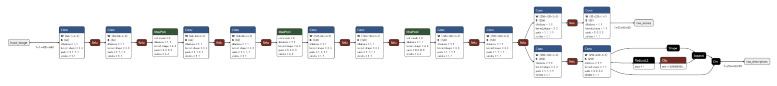
The architecture of the SuperPoint backbone: a VGG-inspired convolutional encoder composed of sequential Conv–ReLU–MaxPool blocks that downsample the input and include a detector head for keypoint probability mapping and a descriptor head for feature extraction.

The backbone of SuperPoint (Figure 5) is a classic Convolutional Neural Network (CNN) design inspired by VGG architecture, which stands out for its effectiveness and simplicity. This architecture has a single main body, called a “shared encoder”, which basically consists of consecutive Conv -> ReLU -> MaxPool blocks. As the input image traverses this linear path, its resolution is halved in each MaxPool layer, and as the layers deepen, more complex features are learned. At the end of this encoder, the structure is divided into two different task-specific “heads”: The first of these, the Detector Head, produces a probability map (raw scores) that indicates which pixels of the image are most likely to be the keypoint. The second branch, the Descriptor Head, creates a high-dimensional mathematical vector (raw descriptors) map that summarizes what these key points and the surrounding pixels look like.

The representative drone image extracted from satellite imagery was compared with each satellite tile. This process was carried out separately on 10 satellite images obtained at different resolution levels (from 500 m to 5000 m). Thus, the matching performance at different altitudes is made comparable. For each tile, the homography matrix was calculated with the RANSAC [41] algorithm over the matching points obtained through the model predictions, and the number of correctly matched (inlier) points was determined. According to the matching successes obtained, the tile with the highest number of inliers was selected and the local coordinates of this region were transferred to the global satellite coordinate system to obtain the best homography matrix ($H_{best}$).

The four corner points of the drone image [(0,0),(1024,0),(1024,1024),(0,1024)] were projected onto the satellite image via this homograph matrix. Thus, the reflection of the drone image on the satellite image and the area it covers are clearly defined. In addition, different match points were projected with this homography matrix and the difference between the actual position and the calculated position of each match was measured. If this difference is less than 5 pixels, the match is classified as “inlier”; otherwise, it is classified as “outlier”. For visual presentation purposes, the projection area of the drone image on the satellite is drawn with a yellow rectangle. Correct matches are shown in red, points that are out of tolerance but remain in the homography area are shown in blue, and all potential matches outside the homograph are shown in green. The tile with the highest inlier rate is highlighted by a bright blue frame. All these markups are visualized in high resolution using the matplotlib library. A scaling matrix (*S*) representing the conversion between the original dimensions of the drone image and its rescaled version at a resolution of 1024×1024 was calculated, multiplied by $H_{best}$ to obtain the full homography matrix ($H_{full}$). This exact homography was used to project the coordinate ($w_{d}/2$, $h_{d}/2$), which is the center point of the drone image, onto the satellite image. The position of this center point on the satellite image, which was obtained as a result of the projection, was determined in pixels. Then, this pixel position was translated into the world coordinate system using the affine transform information in the GeoTIFF file. The reference position information of the drone image was optionally taken from the GeoTIFF file or entered manually by the user in DMS (degrees-minutes-seconds) format. Finally, the geographical distance between these two locations was calculated on the basis of the WGS84 reference system and the spatial accuracy of the system was evaluated based on this distance difference.

## 3. Results

### 3.1. SIFT

In this section, the results of SIFT-based matching operations performed on satellite images with different resolution levels are presented together with visual analysis. The results obtained were evaluated in order to reveal the performance of the system quantitatively and qualitatively at each altitude level.

**Figure 6:**
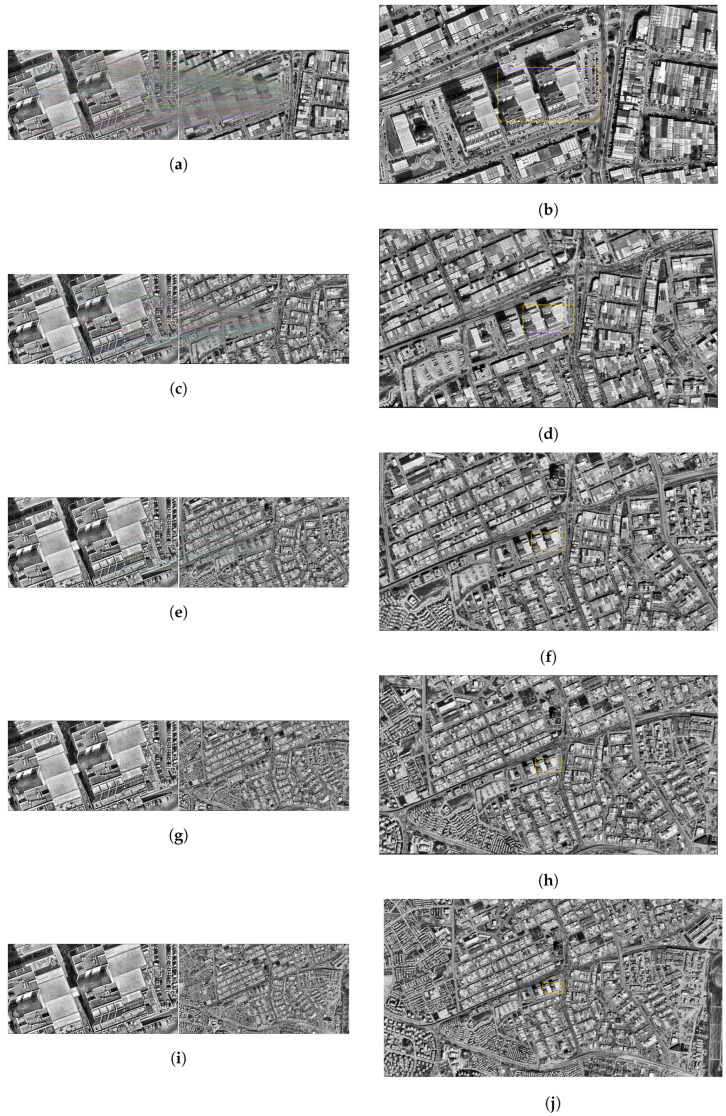
Matching regions on the satellite image obtained from an altitude of (**a**) 500 m, (**c**) 1000 m, (**e**) 1500 m, (**g**) 2000 m, (**i**) 2500 m. The position of the drone on the satellite image of (**b**) 500 m, (**d**) 1000 m, (**f**) 1500 m, (**h**) 2000 m, (**j**) 2500 m (with the SIFT algorithm).

As a result of the matching carried out at this altitude, the number of matching points appears to be high and their distribution to be regular. In particular, the intensified matches along the structural boundaries indicate that the position of the drone is predicted quite accurately in the satellite image as a result of homographic transformation. The location difference is about 0.36 m, ensuring a match rate of over 96%. This situation reveals that the system can give successful results at low altitudes (Figure 6a,b).

At the resolution level of 1000 m, the consistency in the structural regions was maintained, although the number of matching points decreased. The match rate obtained is 92%, and the position estimate was realized with an error of approximately 1.13 m. The area determined on the satellite image is close to the drone location and remains within a meaningful frame (Figure 6c,d).

Some dilution was observed in pairings. Although the placement of the drone image on the satellite still makes sense, there have been slight deviations in the margins. Matching accuracy is calculated as 81%. The position error is just over 1 m. Although this value remains within the operability limits of the system, it indicates that the sensitivity is decreasing (Figure 6e,f).

At this level, the number of matching points has dropped markedly. In the visual data, it is observed that the match lines become irregular and some structures do not contribute enough to the matches. The total match rate is at the level of 61%. The location difference was determined as 2.67 m. This result indicates that the system has started to lose sensitivity (Figure 6g,h).

The accuracy rate obtained as a result of the matching at 2500 m decreased to 41%. It is noteworthy that the matching lines in the images are both small in number and show a random distribution in some regions. The difference between the predicted position and the actual position is about 8.10 m, indicating that the system is beginning to lose its reliability at this level of resolution (Figure 6i,j).

**Figure 7:**
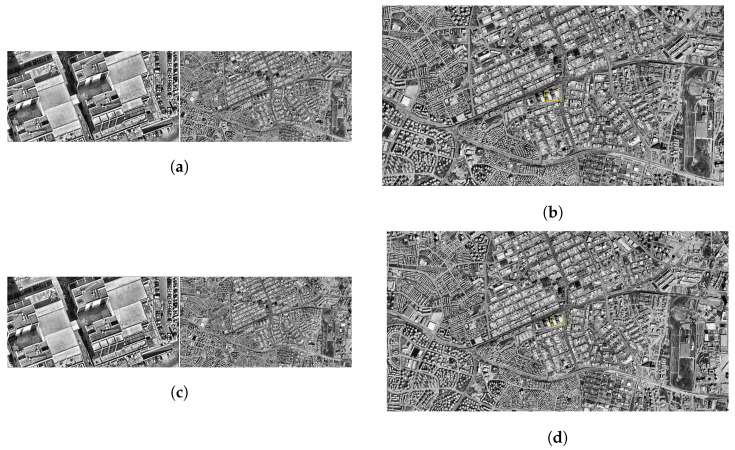

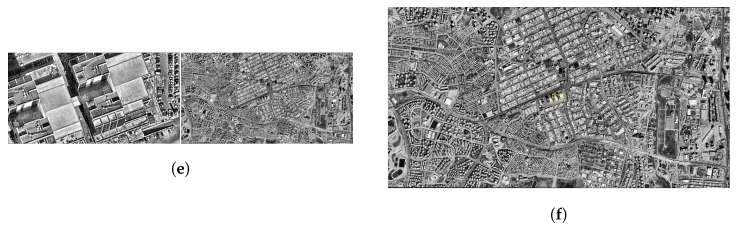
Matching regions on the satellite image obtained from an altitude of (**a**) 3000 m, (**c**) 3500 m, (**e**) 4000 m. The position of the drone on the satellite image of (**b**) 3000 m, (**d**) 3500 m, (**f**) 4000 m (with the SIFT algorithm).

At this altitude, the matching performance is considerably reduced. The lines in the images are weak and inconsistent. The match rate obtained was 21%, and the position error was up to 5.81 m. The visual layout contains a significant deviation. This suggests that match-based positioning loses its precision at high altitudes (Figure 7a,b).

Matching at this level of resolution shows that the number and consistency of matches decreases even more. An accuracy of 21% reveals that the system is not able to adequately maintain the matching structure. Although the position difference of 1.89 m seems low in absolute terms, the clutter in the visual representation suggests that this result may be unstable (Figure 7c,d).

At a resolution of 4000 m, the matching performance of the system is seriously reduced. The match rate obtained is 11%. The lines in the images are sparse and irregular. It is observed that the position of the drone is estimated with an error of about 3.37 m. At this level, the reliability of the system in locating is low (Figure 7e,f).

At this level of resolution (4500 m), the number of matches is limited to only a few points. An accuracy of 4.67% reveals that the match has moved away from the structural foundation. The difference between the predicted drone position and the actual location is about 1969 m. As of this level, it does not seem possible for the system to make match-based location estimation.

Results obtained at the highest resolution level (5000 m) indicate that the system is no longer operable. An accuracy rate of 17% and a position error of 3041.82 m prove that matches have become largely meaningless. The matching lines in the visuals are quite messy and detached from the structural context.

### 3.2. AKAZE

In this section, the results of AKAZE-based matching performed on satellite images with different resolution levels are presented together with visual analysis. The outputs obtained were examined in order to evaluate the performance of the system at each altitude level both quantitatively and qualitatively. Accordingly, the accuracy of the matches obtained, especially at low and medium altitudes, was considered together with the positional consistency between the detected area and the actual drone position.

**Figure 8:**
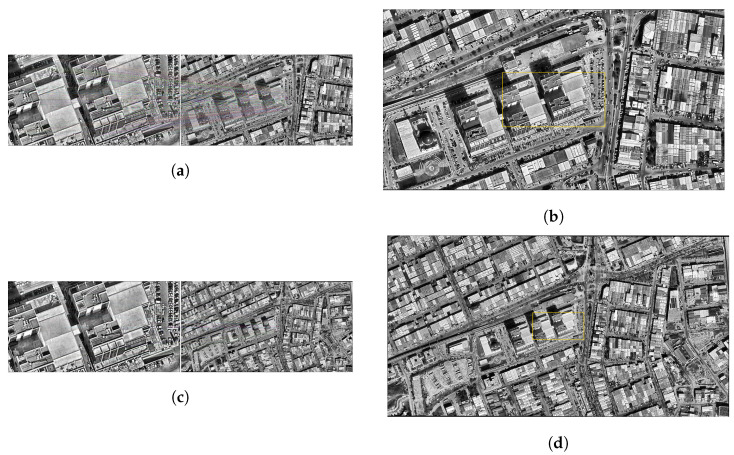
Matching regions on the satellite image obtained from an altitude of (**a**) 500 m, (**c**) 1000 m. The position of the drone on the satellite image of (**b**) 500 m, (**d**) 1000 m (with the AKAZE algorithm).

As a result of the matching performed at an altitude of 500 m, it is seen that the AKAZE algorithm performs very successfully. In visual analysis, the matching lines are concentrated at the apparent structure boundaries, and it is clearly observed that the matching area overlaps with the details in the drone image. The area marked in the satellite image covers an area quite close to the actual midpoint of the drone. The geographical difference calculated according to the log record is only 0.36 m, which shows that the system can operate with millimeter accuracy. In this context, it can be said that the AKAZE algorithm provides reliable matches in high-resolution satellite images (Figure 8a,b).

The matching results obtained at an altitude of 1000 m are also generally successful. The matching lines in the image are mapped to their counterparts on the satellite in a way that is consistent with the structural features in the drone image. The region determined on the satellite is similar to the actual location of the drone image. Looking at the numerical values, the distance between the detected area after the match and the actual drone location was calculated as 1.13 m. This value is still within an acceptable accuracy range for an altitude of 1000 m and shows that the algorithm can work effectively at medium resolution (Figure 8c,d).

When the performance of the AKAZE algorithm at altitudes of 1500 m and above was examined, it was observed that there was a significant decrease in matching accuracy. At 1500 m, the distance between the detected area and the actual location increased to 372.61 m, while at 2000 m this difference increased to 1142.48 m. Similarly, position deviations of 846.69 m occurred at 2500 m, 1259.52 m at 3000 m and 1823.84 m at 3500 m. At even higher altitudes, these deviations reached critical levels. At 4000 m, the error exceeded 13 km and reached 13,284.31 m; at 4500 m, it was 1901.03 m; and at 5000 m, it was 2327.25 m. These results make it clear that as the resolution decreases, the matching success of the AKAZE algorithm drops dramatically and is unable to produce reliable results at high altitude levels. Therefore, this method should only be considered as a valid matching strategy for high-resolution satellite images.

### 3.3. Multi-Template Matching

In this section, the results of the matching operations performed with the Multi-Template Based Normalized Cross-Correlation (Multi-Template NCC) and voting method performed on satellite images with different resolution levels are included. The findings were analyzed in order to evaluate the performance of the system at each altitude level both quantitatively and qualitatively. In this context, the positional harmony between the drone image and the matching zones determined on the satellite image was both visually examined and interpreted by supporting the calculated distance values.

**Figure 9:**
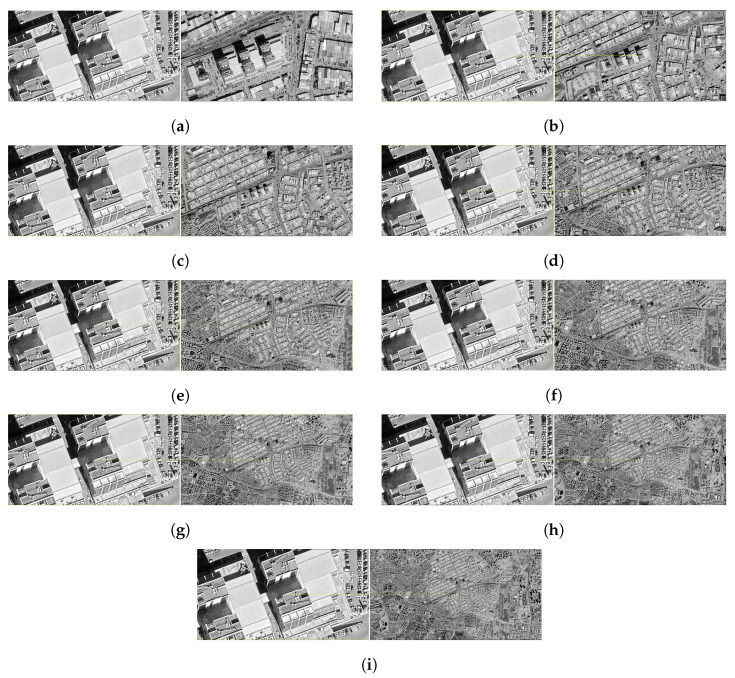
Matching regions on the satellite image obtained from an altitude of (**a**) 500 m, (**b**) 1000 m, (**c**) 1500 m, (**d**) 2000 m, (**e**) 2500 m. (**f**) 3000 m, (**g**) 3500 m, (**h**) 4000 m, (**i**) 4500 m (with the Multi-Template Matching algorithm).

The results obtained at this altitude level (500 m) reveal that the visual match was carried out extremely accurately. The region marked on the satellite image after pairing largely coincides with the area covered by the drone image. The matching lines between the images are symmetrically distributed, and the positioned yellow box successfully captured the drone view. Numerically, the distance between the actual position and the predicted satellite position was calculated to be only 0.43 m. This indicates that the system operates with a fairly high accuracy at low altitude (Figure 9a).

The results obtained at an altitude of 1000 m show a very small deviation compared to 500 m. The visual matching lines still stretch neatly, and the drone region is accurately positioned on the satellite image. The estimated midpoint is located quite close to the center of the visual field, and with a numerical deviation of 2.84 m, it is understood that the system maintains its sensitivity. This supports the stable operation of the method up to the level of 1000 m (Figure 9b).

At this level, the matching accuracy is still sufficient. The obtained satellite coordinates largely coincide with the region corresponding to the drone image. The match lines are homogeneously distributed in the image, and the voting contribution of the templates is prominent. The calculated distance value is 1.69 m, which shows slight differences compared to previous altitudes. This suggests that the matching algorithm also works effectively at mid-range resolutions (Figure 9c).

In the match made at an altitude of 2000 m, the visual integrity was not disturbed, and the drone area determined by voting was successfully marked on the satellite image. Under the influence of the multi-template approach, different variations contributed to the match lines, and the prediction point largely coincided with the target region. The calculated distance value is 1.80 m, resulting in a low deviation, similar to previous altitudes. In this context, it can be said that the system shows high stability at resolutions up to 2000 m (Figure 9d).

At this altitude, a partial deviation in system performance was observed. Visual matching lines still establish a consistent relationship between the drone and satellite regions, but are spread over a wider area than at previous altitudes. It is noticeable that the yellow box does not coincide with the center and remains on the outer borders. The distance value was calculated as 11.77 m, which indicates a margin of error approaching the tolerance limit for the system (Figure 9e).

At this altitude level, the overall matching success of the system exhibits a stability close to previous levels. In the image, the matching lines show a homogeneous distribution in terms of density, and a significant overlap is observed between the drone image and the satellite region. The yellow frame comprehensively surrounds the target area, with the matching lines clustered pointwise in the center. The numerically obtained distance value of 5.62 m provides an acceptable error rate for medium resolution satellite data (Figure 9f).

As a result of the pairing at 3500 m, there was a noticeable deviation between the predicted zone and the actual drone position. In the image, the matching lines are spread over a wide area and the intensity is significantly reduced. The yellow box is positioned slightly deviated from the satellite area corresponding to the drone image. The distance value was calculated as 11.26 m, which indicates that the system can produce less accurate results at this altitude. Nevertheless, the overall structure has been preserved, and the estimated area remains in close proximity to the drone (Figure 9g).

Contrary to expectations, the results observed at this altitude showed a positive turn. The lines in the visual matches are prominently centered, despite the high-resolution satellite image. The box marked on the satellite is located very close to the area where the drone image corresponds, and the visual integrity is preserved. The measured distance value was reduced to a low level of 3.56 m. This result shows that at an altitude of 4000 m, the matching success has rebounded and the stability has increased (Figure 9h).

When 4500 m was reached, a slight deterioration in the overall performance of the system was observed. Although the yellow box marked on the satellite is located close to the position of the drone, there is a slight irregularity in the matching lines and a decrease in density. This shows that the visual similarity is reduced and the decision process of the matching algorithm becomes more complex. However, the calculated distance value of 3.29 m reveals that the system still provides an acceptable level of accuracy for this altitude (Figure 9i).

When the results of the system at the highest altitude are examined, it is noticed that the visual matching success is significantly reduced. The matching lines are widely distributed and centralization is reduced. The yellow box on the satellite image has moved away from the area of the drone image where it should be. The measured distance value was recorded as 21.69 m, the highest deviation among all tested levels. This indicates that methodological limitations become more pronounced at high altitude levels and indicate that the sensitivity of the system decreases after this limit.

### 3.4. Deep Learning-Based Feature Matching Algorithms

#### 3.4.1. LoFTR

In the following figures, matching operations were carried out between satellite images obtained at different altitudes and representative drone images using the LoFTR algorithm. As a result of each match, the midpoint of the drone image projected onto the satellite was determined with the help of a homography matrix and this point was converted into a real-world coordinate system using the transformation information in the GeoTIFF file. This location information obtained by the system was then compared with the reference point manually entered by the user.

The real-world coordinates of the representative drone image, which are fixed in all images, are defined as latitude (Y) = 39.969539 and longitude (X) = 32.745036. The geographical distance of the projection center to this fixed reference point obtained as a result of each match was calculated in meters and thus the positional accuracy performance of the system was evaluated quantitatively.

**Figure 10:**
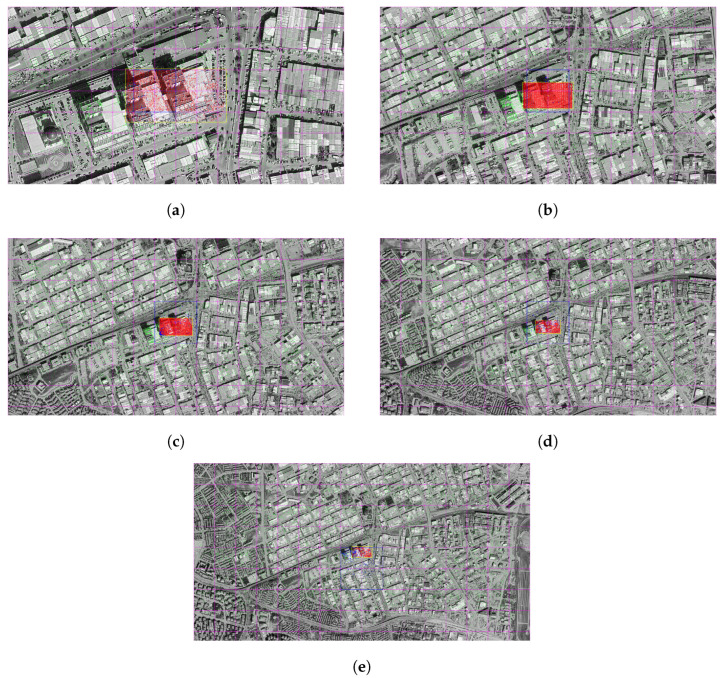
Matching regions on the satellite image obtained from an altitude of (**a**) 500 m, (**b**) 1000 m, (**c**) 1500 m, (**d**) 2000 m, (**e**) 2500 m (with LoFTR algorithm).

As a result of this matching, the projection center of the representative drone image on the satellite was determined at approximately (32.745033, 39.969543) and it was calculated that there was only a 0.56 m difference between it and the actual center. Out of 4407 matching points, 4239 were classified as inlier, thus achieving a high matching accuracy of 96.2% (Figure 10a).

Z In the analysis performed at 1000 m, the center point of the projection was calculated as (32.745037, 39.969559) and the distance of this position from the reference center was measured as 2.20 m. The number of matches is quite high: 8252 out of 8319 points were considered correct matches, resulting in an extremely strong inlier performance of 99.2% (Figure 10b).

At this altitude, the position of the projection center was determined as (32.745037, 39.969543). The difference to the true center was only 0.51 m, and 4247 of the 4518 matches were found to be inlier. This indicates that the system matches with 94.0% accuracy (Figure 10c).

The matching point obtained from the satellite data coincided with the coordinate (32.745010, 39.969601) and a deviation of 7.20 m occurred compared to the reference position. Out of a total of 1460 matches, 1204 were evaluated as correct and an inlier rate of 82.5% was obtained (Figure 10d).

This time, the projection center fell to (32.745049, 39.969479). A distance of 6.78 m was calculated between this location and the actual center. However, the match quality dropped significantly, with only 441 out of 812 matches counted as correct matches. At this altitude, the inlier rate decreased to 54.3% (Figure 10e).

**Figure 11:**
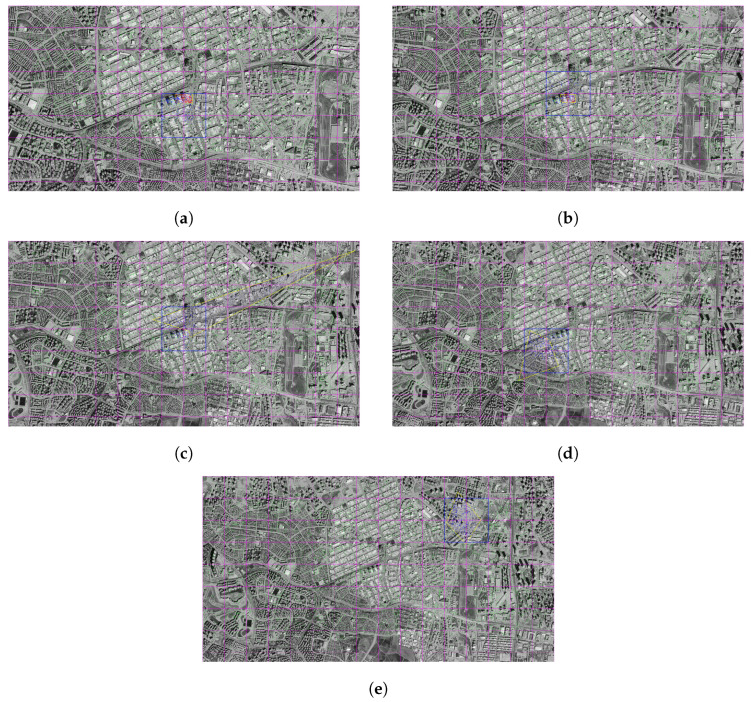
Matching regions on the satellite image obtained from an altitude of (**a**) 3000 m, (**b**) 3500 m, (**c**) 4000 m, (**d**) 4500 m, (**e**) 5000 m (with LoFTR algorithm).

The matched point was found at approximately (32.745049, 39.969517) and the distance to the reference center was measured as 2.68 m. However, in total, only 177 correct matches were obtained out of 547 matches, which corresponds to a low success rate of 32.4% (Figure 11a).

The projection point is located at the coordinate (32.745056, 39.969528). A deviation of 2.08 m occurred between it and the true center, while only 79 of the 365 matches were identified as inlier. Accordingly, the inlier rate decreased to 21.6% (Figure 11b).

In the matching made at this altitude, the projection point corresponded to the coordinate (32.744949, 39.969559) and although a deviation of 7.73 m was detected between it and the reference center, only 13 out of 319 matches were considered correct matches and the inlier rate remained at 4.1%. For this reason, as a result of the homography obtained, it was seen that the projected image area was far from representing the geometric structure of the real drone image (Figure 11c).

The resulting projection center was located at (32.740955, 39.967247) and deviated from the reference point at a considerable distance of 431.63 m. Out of a total of 369 matches, only 13 were considered correct, resulting in a low success rate of 3.5%. Moreover, in this match, the area of the drone image deviated from the expected rectangular form when projected onto the satellite image (Figure 11d).

In matching with the satellite image taken from the farthest altitude, the projection midpoint was calculated at the coordinate (32.760643, 39.978264) and the difference between it and the true center was a very serious deviation of 1648.04 m. Out of 328 matches, only 10 were classified as inlier, corresponding to an extremely low accuracy rate of 3.0%. However, it was observed that the projected image area did not correspond to the original geometric structure of the drone image (Figure 11e).

#### 3.4.2. SuperPoint and SuperGlue

In the images below, satellite images taken from different altitudes and representative drone images are matched using SuperPoint and SuperGlue algorithms. After each pairing, the center point of the drone image was projected onto the satellite image by homography transformation.

This position, which was obtained as a result of the projection, was compared with the previously known reference point, and the accuracy of the system’s position estimation at each altitude was evaluated numerically.

**Figure 12:**
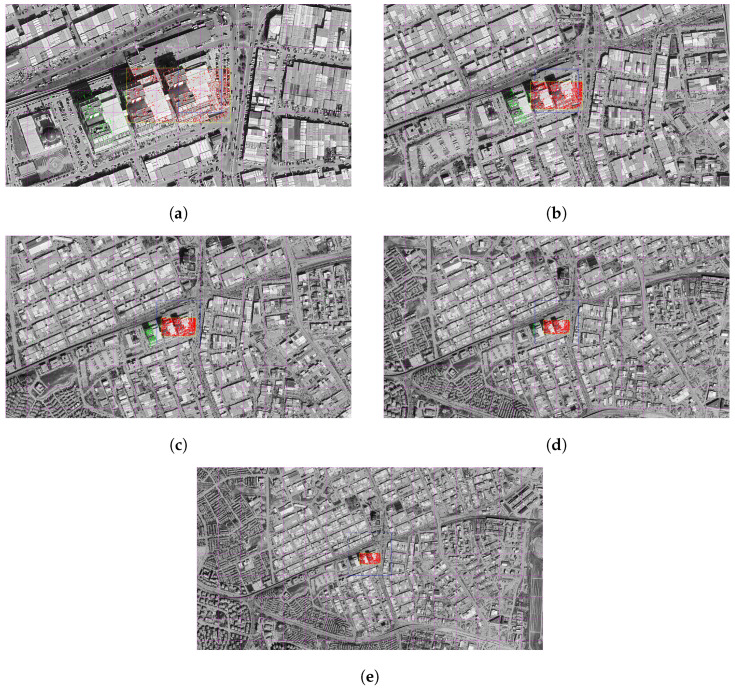
Matching regions on the satellite image obtained from an altitude of (**a**) 500 m, (**b**) 1000 m, (**c**) 1500 m, (**d**) 2000 m, (**e**) 2500 m (with Superpoint and SuperGlue algorithm).

The resulting projection center was reduced to (32.745036, 39.969543) and the distance to the actual reference point was measured as only 0.44 m. Out of a total of 2753 matches, 2364 were evaluated as inliers, resulting in a high match success rate of 85.9% (Figure 12a).

It was determined that there was a difference of 2.13 m between the projection center calculated at the coordinate (32.745037, 39.969558) and the reference center. A high accuracy was achieved at this altitude, and 1834 of 1874 matches were considered valid, reaching an inlier rate of 97.9% (Figure 12b).

The distance between the point (32.745038, 39.969545) and the actual center obtained as a result of the projection process is calculated as 0.67 m. Out of 1229 matches, 1209 were considered correct matches, an impressive 98.4% of the time (Figure 12c).

At this altitude, the projection point was determined as (32.745005, 39.969598) and the distance to the reference point was measured as 7.10 m. The match quality is again quite high. Of the 690 matches, 674 were classified as inliers, with an accuracy of 97.7% (Figure 12d).

The projection point formed on the satellite image is located at the coordinate (32.745044, 39.969480) and is 6.54 m away from the actual center. In this matching, 442 out of 447 matches were found to be valid and a very accurate result was obtained with an inlier rate of 98.9% (Figure 12e).

**Figure 13:**
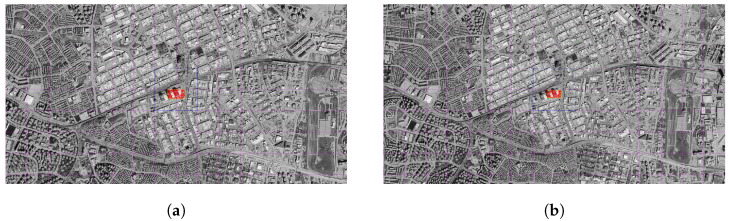

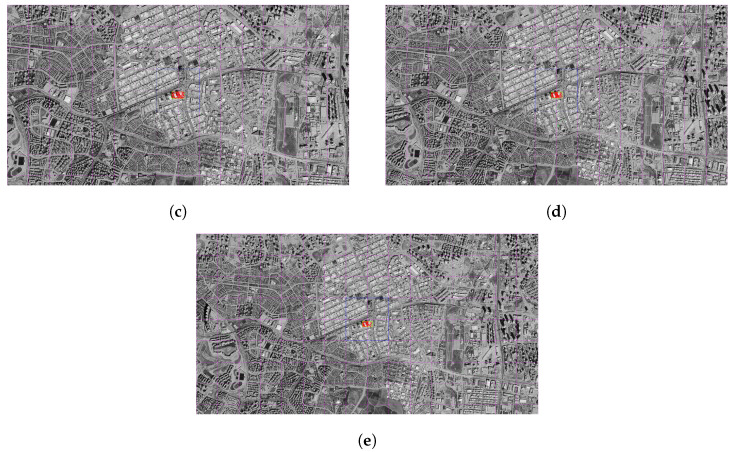
Matching regions on the satellite image obtained from an altitude of (**a**) 3000 m, (**b**) 3500 m, (**c**) 4000 m, (**d**) 4500 m, (**e**) 5000 m (with Superpoint and SuperGlue algorithm).

The center point of the paired projection was calculated as (32.745044, 39.969521), and the distance from the reference center was determined as 2.07 m. Of the 329 matches, 328 were deemed valid, recording an outstanding inlier rate of 99.7% (Figure 13a).

The projection obtained in this analysis fell on the point (32.745053, 39.969536) and measured the distance from the true center as 1.45 m. Out of a total of 238 matches, 231 were classified as inliers, resulting in a matching accuracy of 97.1% (Figure 13b).

The projection center was determined at the coordinate (32.745045, 39.969584), and the distance from this point to the actual reference position was calculated as 5.02 m. Of the 187 matches, 182 were evaluated as correct matches and 97.3% inlier success was achieved (Figure 13c).

Corresponding to the projection center position (32.745005, 39.969560), determined as a result of image matching, the distance between the reference center and the reference center was found to be 3.52 m. In this matching, 147 out of 153 matches were accepted as valid, resulting in an accuracy of 96.1% (Figure 13d).

In the analysis performed at the highest altitude, the projection center was calculated as (32.745062, 39.969488) and this point was determined to be 6.04 m from the center. Of the 108 matches, 99 were marked as correct matches, and the system was able to show a strong match accuracy with an inlier rate of 91.7% (Figure 13e).

## 4. Discussion

In this study, the accuracy of position matches performed at different satellite image resolutions was evaluated comparatively with both conventional and CNN-based methods. The analyses quantitatively reveal the performance of each algorithm at different altitude levels through position deviation (in meters) Table 1.

At low altitudes (500–1000 m) all methods showed high accuracy with a deviation value of less than 3 m. At this level, the LoFTR and SIFT algorithms, in particular, stood out with a deviation of less than 1 m. AKAZE and NCC+Voting methods showed similar performance. This suggests that both traditional and learning-based approaches provide sufficient match quality for images with a low resolution difference.

At medium altitudes (1500–3000 m), the differences between the methods are pronounced. AKAZE showed serious distortions in this altitude range. For example, at 2000 m, it lost its reliability with a deviation of 1142.48 m. On the other hand, the NCC + Voting method has produced stable results by maintaining low deviation values with its structure based on the principle of template diversity and majority vote. Learning-based approaches such as SuperPoint+SuperGlue and LOFTR have gained superiority over traditional methods, especially in the 2500–3000 m band, with deviation values below 10 m.

At high altitudes (3500–5000 m), it has been observed that deviations increase. AKAZE and SIFT produced deflections of over 1000 m. This reveals that solubility differences and artificial deformations adversely affect the success of traditional methods. At this point, LOFTR outperformed all other methods with a deviation of 15.83 m at 5000 m, followed by SuperPoint + SG in second place with a deviation of 25.18 m. The NCC + Voting method, on the other hand, has largely maintained its low deviation and has offered a more robust result than some traditional methods.

As a result, although traditional methods perform satisfactorily at low and medium altitudes, it has been observed that deep learning-based algorithms (especially LOFTR) give more stable and successful results as altitude increases. However, methods such as NCC + Voting, which do not involve machine learning but incorporate diverse decision-making, have also delivered remarkably stable performance at high altitudes. This analysis reveals that the choice of method according to different operational altitudes should be made carefully and that deep learning-based systems are more advantageous, especially with high resolution differences.

This study, which carried out a simulation for real-time applications, is useful for testing algorithms in an isolated environment, but does not reflect the challenges of real-world sensor data (noise, atmospheric effects, etc.). The most important step for future studies will be verification with real UAV and satellite data.

The scope of the study focuses on scale change, so factors such as rotation and illumination were not tested. This prevents us from demonstrating the full capabilities of algorithms like SIFT, but it is a necessary simplification to test our hypothesis.

The homography matrix is a geometrically simple model. However, this choice was chosen in line with the long-term goal of the study: to develop a real-time and embedded system (using Vitis AI on FPGA). Homography is considered suitable for hardware acceleration and parallelization thanks to matrix operations, while more complex models can bottleneck real-time performance.

Our system alone generates 2D (latitude/longitude) position, but this is not an incomplete solution. It aims to easily obtain a full 3D position solution by combining the altitude data to be obtained from the barometric altimeter sensor in a standard UAV with the output of our system.

## 5. Conclusions

In this study, the matching processes performed between satellite images with different resolution levels and drone-borne images were evaluated and various traditional and deep learning-based methods were compared. The main objective is to determine the accuracy of alternative positioning systems in scenarios where GPS signals cannot be used.

As a result of the analyses performed, it was determined that all methods can match with high accuracy at low altitudes (500–1000 m). However, as the altitude increases, it has been determined that traditional approaches lose performance. In particular, methods such as AKAZE and SIFT have achieved serious deviation values at high altitudes (3500 m and above). In contrast, learning-based methods such as SuperPoint+SuperGlue and LOFTR were able to produce more stable and low-deviation results despite increasing resolution differences and geometric aberrations.

In addition, it has been observed that hybrid and voting-based traditional algorithms, such as the NCC+Voting method, are less affected by altitude changes and show a very consistent performance by taking advantage of the variety of templates. This shows that visual-based positioning systems can be effectively developed not only with methods based on deep learning, but also with classical approaches enriched with appropriate pre-processing and decision mechanisms.

The results obtained revealed that both traditional and deep learning-supported matching methods can be configured and used correctly in operational scenarios where GPS signals are blocked or unreliable. In this context, it is considered that additional modules such as multi-scale matching, depth estimation and geographic foresight integration can further improve system performance in future studies.

## Figures and Tables

**Table 1:** Drone position deviation comparison (in meters) found relative to conventional and CNN-based methods. Bold indicates the best data of each altitude.

Altitude (m)	SIFT	AKAZE	NCC + Voting	LoFTR	SuperPoint + SG
500	**0.36**	**0.36**	0.43	0.56	0.44
1000	**1.13**	**1.13**	2.84	2.20	2.13
1500	1.07	372.61	1.69	**0.51**	0.67
2000	2.67	1142.48	**1.80**	7.20	7.10
2500	8.10	846.69	11.77	6.78	**6.54**
3000	5.81	1259.52	5.62	2.68	**2.07**
3500	1.89	1823.84	11.26	2.08	**1.45**
4000	**3.37**	13,284.31	3.56	7.73	5.02
4500	1969.00	1901.03	**3.29**	431.63	3.52
5000	3041.82	2327.25	21.69	1648.04	**6.04**

## Data Availability

The data presented in this study are available in: https://github.com/MetaversePrime/Multi-Template-NCC-Geolocalization (accessed on 2 November 2025). https://github.com/MetaversePrime/AKAZE-Geo-Localization (accessed on 2 November 2025). https://github.com/MetaversePrime/SIFT-FLANN-Geo-Localization (accessed on 2 November 2025). https://github.com/reader313/LofTR-and-SuperGlue-Superpoint-Geolocalization (accessed on 2 November 2025).

## Abbreviations

The following abbreviations are used in this manuscript:

SIFT	Scale-Invariant Feature Transform
AKAZE	Accelerated KAZE
NCC	Normalized Cross-Correlation
SuperPoint	Self-Supervised Interest Point Detector and Descriptor
LoFTR	Detector-Free Local Feature Matching with Transformers
UAV	Unmanned Aerial Vehicle
RANSAC	Random Sample Consensus
GNSS	Global Navigation Satellite System
GPS	Global Positioning System
IMU	Inertial measurement unit
VIO	Visual-inertial odometry
VPR	Visual location recognition
JPEG	Joint Photographic Experts Group
DMS	Degree-minute-second
CLAHE	Contrast Limited Adaptive Histogram Equalization
DoG	Difference of Gaussians

FLANN	Fast Library for Approximate Nearest Neighbors
MLDB	Modified Local Difference Binary
CNN	Convolutional Neural Network
